# Cell-Mediated Drug Delivery: From Carriers to Therapeutics

**DOI:** 10.3390/pharmaceutics18070890

**Published:** 2026-07-20

**Authors:** Lisa Gherardini, Monia Taranta

**Affiliations:** Istituto di Fisiologia Clinica (IFC), Consiglio Nazionale delle Ricerche (CNR), 53100 Siena, Italy

Nanomedicine has profoundly transformed the delivery of therapeutic agents by improving drug pharmacokinetics, protecting labile cargos, and expanding the therapeutic potential of a wide range of molecules. Nevertheless, despite substantial advances in carrier engineering, efficient tissue targeting, penetration of biological barriers, and durable therapeutic efficacy remain major challenges, particularly in oncology and in diseases affecting anatomically impervious districts [1]. In this context, cell-mediated drug delivery has emerged as one of the most promising strategies for overcoming these limitations by exploiting the intrinsic biological properties of living cells and cell-derived components, including active homing, immune compatibility, and the ability to interact dynamically with the pathological microenvironment [2,3].

One of the defining challenges in cell-mediated drug delivery is no longer the identification of suitable therapeutic cargos, but rather the development of delivery systems capable of transporting those cargos to the right site at the right time, and in sufficient amounts, to achieve a therapeutic effect. Although remarkable advances have been made in carrier engineering, biodistribution and drug availability remain among the major obstacles to clinical translation, particularly for tumors located in difficult-to-reach tissues such as the central nervous system [4].

Beyond presenting recent advances, this Special Issue provides a snapshot of a field undergoing a profound conceptual transformation that is reshaping the development of next-generation delivery systems. It brings together complementary perspectives that span cell biology, biomimetic engineering, synthetic biology, and programmable cellular therapies, while maintaining a constant focus on the translational challenges that continue to limit clinical implementation. The collection reveals an integrated landscape in which living cells, extracellular vesicles, biomimetic materials, and engineered nanoplatforms are progressively designed to become advanced therapeutic systems.

Consistently, these studies indicate that successful therapeutic delivery depends not only on the physicochemical properties of the carrier—such as its targeting selectivity and cargo-loading capacity—but also on therapeutic efficacy, which is ultimately determined by the ability of the delivery system to navigate the in vivo environment, accumulate at the intended pathological site, and preserve its biological activity. Thus, despite remarkable advances in delivery technologies, achieving effective biodistribution remains one of the major barriers to clinical translation.

Finally, this set of contributions provides a comprehensive overview of both the promise and the challenges of exploiting biological carriers for targeted therapeutic delivery. While living cells and cell-derived vesicles benefit from intrinsic mechanisms that enable selective tissue homing, their clinical application remains constrained by off-target sequestration, biological variability, and the limited control over their in vivo biodistribution and fate. Addressing these challenges will be essential to fully harness the therapeutic potential of cell-based delivery systems. Tekin et al. provide compelling evidence of homotypic targeting through the preferential accumulation of radiolabeled tumor cells in tumors sharing the same cellular origin, while simultaneously highlighting significant sequestration within the lungs and reticuloendothelial organs. Similarly, Kono et al. demonstrate that mesenchymal stem cells can significantly improve the tumor accumulation and therapeutic efficacy of doxorubicin-loaded liposomes, although the biological complexity of these carriers continues to influence biodistribution, persistence, and treatment outcome, indicating that the intrinsic targeting properties of biological systems represent a unique therapeutic opportunity, but their successful clinical exploitation will require greater control over their biological behavior and reproducibility.

Extracellular vesicles (EVs) have emerged as one of the most promising biological delivery platforms, while at the same time exemplifying many of the challenges that continue to limit clinical translation. As highlighted by Mohak and Fabian, EVs combine biocompatibility, low immunogenicity, and efficient cargo transport across biological barriers, making them attractive delivery vehicles. However, challenges related to manufacturing, heterogeneity, and quality control continue to limit their clinical translation. Addressing these limitations, Pourali et al. demonstrate the potential of hybrid EV-based nanoplatforms for targeted RNA delivery and in vivo gene silencing, highlighting the promise of integrating natural and engineered delivery systems. More broadly, these studies reflect a growing convergence between naturally derived and engineered EV-based delivery systems, where biological specificity is increasingly complemented by the tunability and scalability offered by nanotechnology [5,6].

Another emerging theme of this Special Issue is the expansion of cell-mediated delivery beyond oncology, with applications extending to inflammatory diseases, chronic wounds, and regenerative medicine, as discussed by Shqair and colleagues. In parallel, biomimetic strategies are being developed to preserve the biological advantages of cells while overcoming their practical limitations. As reviewed by Kim et al., cell membrane-coated nanoparticles combine immune evasion, prolonged circulation, and targeting capabilities with the scalability of synthetic materials, representing a promising bridge between nanomedicine and live-cell therapies.

Perhaps the most important conceptual advance reflected throughout this collection is the transition from viewing cells simply as passive transport vehicles to engineering them as active therapeutic systems that can sense pathological signals, integrate environmental information, and execute programmable therapeutic responses with an unprecedented level of complexity. As discussed by Mashele and Gherardini et al., this evolution can transform cells into dynamic therapeutic platforms capable of adapting their activity to the disease microenvironment; however, this introduces new challenges such as manufacturing consistency, standardization, quality control, regulatory oversight, biosafety, and long-term control of engineered cellular behavior, which are becoming equally important determinants of successful clinical translation.

The evolution of cell-mediated drug delivery will therefore depend not only on technological innovation but also on the establishment of robust manufacturing, quality control, and regulatory frameworks capable of supporting increasingly complex biological therapies. Taken together, the contributions in this Special Issue depict a field moving beyond the development of individual delivery vehicles toward the rational integration of complementary biological and engineering strategies. Hybrid systems integrating engineered EVs, biomimetic nanoparticles, and programmable cellular therapies hold great promise to overcome current limitations in targeting, biodistribution, and therapeutic control. The future of cell-mediated delivery is unlikely to be defined by a single optimal platform; rather, it will emerge from the combination of the intrinsic biological capabilities and the opportunities for scalability and tunability of engineered materials. From this perspective, this Special Issue not only reflects the current maturity of the field but also highlights the principles that will guide its future developments.
